# A generalized equation for the calculation of receptor noise limited colour distances in *n*-chromatic visual systems

**DOI:** 10.1098/rsos.170712

**Published:** 2017-09-20

**Authors:** R. C. Clark, R. D. Santer, J. S. Brebner

**Affiliations:** 1 School of Natural and Computing Sciences, University of Aberdeen, King's College, Aberdeen AB24 3UE, UK; 2 Institute of Biological, Environmental, and Rural Sciences, Aberystwyth University, Aberystwyth SY23 3FG, UK

**Keywords:** colour discrimination, colour perception, chromatic contrast, animal vision, photoreceptor

## Abstract

Researchers must assess similarities and differences in colour from an animal's eye view when investigating hypotheses in ecology, evolution and behaviour. Nervous systems generate colour perceptions by comparing the responses of different spectral classes of photoreceptor through colour opponent mechanisms, and the performance of these mechanisms is limited by photoreceptor noise. Accordingly, the receptor noise limited (RNL) colour distance model of Vorobyev and Osorio (Vorobyev & Osorio 1998 *Proc. R. Soc. Lond. B* **265**, 351–358 (doi:10.1098/rspb.1998.0302)) generates predictions about the discriminability of colours that agree with behavioural data, and consequently it has found wide application in studies of animal colour vision. Vorobyev and Osorio (1998) provide equations to calculate RNL colour distances for animals with di-, tri- and tetrachromatic vision, which is adequate for many species. However, researchers may sometimes wish to compute RNL colour distances for potentially more complex colour visual systems. Thus, we derive a simple, single formula for the computation of RNL distance between two measurements of colour, equivalent to the published di-, tri- and tetrachromatic equations of Vorobyev and Osorio (1998), and valid for colour visual systems with any number of types of noisy photoreceptors. This formula will allow the easy application of this important colour visual model across the fields of ecology, evolution and behaviour.

## 1. Background

Colour sensations are constructed by nervous systems via comparisons of the responses of different spectral classes of photoreceptor [1]. Across taxa there is considerable variation in the number of spectral classes of photoreceptor in the retina, and in the wavelengths of light to which these different spectral classes respond most strongly [1–3]. Consequently, animal colour sensations can differ substantially to our own, yet it is essential in many fields of enquiry to evaluate similarities and differences in colour from an animal's eye view.

The responses of an animal's photoreceptors to a particular stimulus can be computed, given the reflectance spectrum of that stimulus and its background, the spectral sensitivity functions of the animal's photoreceptors, and the spectrum of illuminant light [1,4,5]. The calculated responses of the relevant classes of photoreceptor can then be used to determine the position of the stimulus within that animal's colour space [1,6–9]. Often of interest is the question of whether particular colour loci can be discriminated from one another, and key to this issue is the idea that the performance of colour opponent mechanisms is limited by photoreceptor noise [4,10,11]. The receptor noise limited (RNL) model allows the discriminability of colours to be assessed in a way that accounts for photoreceptor noise [4,10,11] and consequently is among the most influential and well-used tools in studies involving animal colour vision.

The original publication of the RNL model provides equations allowing colour distances to be determined for di-, tri- and tetrachromatic visual systems [4]. Most animals with the capability of colour vision do indeed appear to be functionally di-, tri- or tetrachromatic, and theoretical analyses also suggest that such visual systems are adequate for extracting the information available in natural spectra. However, many organisms have photoreceptor machinery that could mediate more complex colour vision, even if the mechanisms employed for a particular behavioural task can be shown to be di-, tri- or tetrachromatic. For example in butterflies, screening pigments produce a wide variety of spectral types of receptor that varies among families, species and even sexes, with the extreme so far discovered being 15 spectral types of photoreceptor in *Graphium sarpedon* [12,13]. In spite of the potential for greater complexity, foraging in *Papilio xuthus* relies on a tetrachromatic mechanism [14], and oviposition behaviours in *Papilio aegeus*, *Pieris brassicae*, and *Pieris rapae* are adequately explained by tri- or tetrachromatic mechanisms [15,16]. Dragonflies have an extraordinary diversity of opsin genes whose expression varies with life stage and eye region, with more than 10 visual opsins expressed in the adult eye of some species [17], and their involvement in colour-driven behaviour is currently unknown; and the 12 spectral receptor classes serving colour vision in mantis shrimp operate as an unusual scanning colour recognition system unlike any other known colour visual mechanism [18]. For such organisms, the experimenter may wish to test the plausibility of any possible *n*-chromatic visual mechanism if the photoreceptors involved in a particular behavioural act are unknown or in doubt (c.f. [14,19]).

In other study species, visual mechanisms have the potential to be more complex than has been assumed, necessitating analysis of *n*-chromatic visual mechanisms. For example, in true flies five spectral classes of receptor are present through the majority of the retina [20,21]. Although it has often been assumed that colour discrimination relies only on the four classes of long visual fibre [21,22], evidence from *Drosophila* shows that the short visual fibres, previously assumed to serve achromatic functions only, contribute to colour discrimination [23]. Among the Tabanidae and Dolichopodidae, differently coloured lenses in different regions of the compound eye shift the sensitivities of photoreceptors within their ommatidia [24–27], raising the possibility that still more functional classes of receptor may exist. Outside of the insects, the double cones of fishes, reptiles, and birds are also assumed to serve achromatic tasks only, but there is evidence from blackbar triggerfish that they can contribute to colour discrimination in that species [28]. Thus, there may be a need for experimenters on such systems to consider *n*-chromatic visual mechanisms during their investigations.

In order to apply the RNL model to colour visual systems hypothesized to involve greater than four photoreceptors, experimenters have had to derive the relevant RNL model equations (such as those provided for pentachromatic and hexachromatic colour visual systems by [19]). Thus, to simplify the application of the RNL model to colour visual systems of any dimensionality, we derive a single equation that can be used to compute RNL colour distances for *n*-chromatic visual systems.

## 2. Basic assumptions

We begin with the fact that for any animal with the capability, colour stimuli are described by photoreceptor responses. The quantum catch of a photoreceptor, *q*, is computed by

2.1 $$q_{i}=r_{i}\int_{\lambda }S_{i}(\lambda )I_{s}(\lambda )\;\text{d}\lambda ,$$

where *q_i_* is the quantum catch of receptor *i*, *S_i_*(*λ*) is its spectral sensitivity function and *I_s_*(*λ*) is the spectrum of light entering the eye from a stimulus of interest, *s*. *λ* is the wavelength and *r_i_* is the range sensitivity factor, through which photoreceptor responses are adjusted to the spectrum of light entering the eye from the visual background, *I*_b_:

2.2 $$r_{i}=\frac{1}{\int_{\lambda }S_{i}(\lambda )I_{b}(\lambda )\;\text{d}\lambda }.$$

The process of phototransduction leads to a non-linear relationship between quantum catch, *q_i_*, and photoreceptor response, *f_i_* [1,5,9]. The RNL model can be applied to linearly or non-linearly transformed photoreceptor responses and the two are equivalent for stimuli that are close to a reference [4,10,11].

We will assume that there are *N* receptors and that the output of receptor *i* has random noise with variance $e_{i}^{2}$ [4]. We follow the notation of Vorobyev *et al*. [11] and the usual assumptions [4] that:

1. for a visual system with *N* receptor channels, colour is encoded by *N *− 1 opponent mechanisms and the achromatic signal is discarded;
2. opponent mechanisms give zero signal for stimuli that differ from background in intensity only; and
3. thresholds are set by receptor noise, and not by opponent mechanisms.

The response of the *N* receptors to a stimulus can be represented by a column vector ***f*** where $f={\left(\;f_{1},f_{2},\ldots ,f_{N}\right)}^{T}$*.* (Superscript *T* indicates ‘transpose’*.*) Similarly, the output of the (*N *− 1) opponent mechanisms can be represented by a column vector ***x*** with (*N − *1) components. It is also assumed [4,10,11] that ***x*** depends linearly on ***f***. Hence the relationship between the two vectors can be written mathematically as

2.3 x=Ff,

where ***F*** is an (*N *− 1) *×* *N* matrix with constant components.

As pointed out by Vorobyev & Osorio [4], assumption 2 imposes a constraint on the form of the matrix ***F***. They show (their equation A8) that the sum of the components in each row of ***F*** must be zero. They consequently choose ***F*** to be the matrix which defines each *x_i_* by

2.4 $$x_{i}=f_{i}-f_{i+1}$$

for i=1,…(N−1)*.* As we wish to generalize the expressions published by Vorobyev & Osorio [4], we will follow their choice. No scaling factor is required in this equation as the colour distance is invariant under changes of the scale of each *x_i_* (see below).

Equation (2.4) implies that the outputs of the opponent mechanisms depend only on the differences between the outputs of the photoreceptors. It can be shown generally that any matrix ***F*** satisfying equation A8 in [4], and thus assumption 2, must have this property. What equation (2.4) does not do, is uniquely define the opponent mechanism outputs. Any linear combination of the differences ( *f_i_ *− *f_j_*) is a possible choice of an opponent mechanism output satisfying assumption 2 and that choice will not affect the colour distance Δ*S* defined by Vorobyev & Osorio [4]. It is in this limited sense that the statement in assumption 3, that thresholds are not set by opponent mechanisms, is valid.

## 3. The Mahalanobis distance

We need to have a measure of the difference between the responses to two different stimuli. Let two responses be ***f*** and ***g*** with corresponding vectors ***x*** and ***y*** after the opponent mechanisms. Let Δf=( f−g) and Δx=(x−y). Vorobyev & Osorio [4] suggested that in the *N *− 1 dimension space of ***x*** vectors**,** the distance between the two stimuli could be defined by Δ*S* where the square of Δ*S* is given by

3.1 $${\left(\Delta S\right)}^{2}={\Delta x}^{T}R^{-1}\Delta x.$$

Here ***R****^−^*^1^ is the inverse of ***R***, the variance-covariance matrix of the variables *x_i_*_._

The distance defined in equation (3.1) is known to statisticians as the Mahalanobis distance [29] after the Indian statistician who first used it. Note that although Δ*S* is referred to as a distance, it is actually a pure number as it is the ratio of the ‘distance’ between the two stimuli to the generalized standard deviation of the distribution. It gives the equivalent, in any number of dimensions, of the one-dimensional statement that two data points are Δ*S* standard deviations apart. Since it is a number, it has the property of ‘scale invariance’ which means that each *x_i_* can be rescaled without changing Δ*S.* It is also invariant under rotations of the axes in the space of the vectors ***x***. This gives us the freedom to choose the opponent mechanisms' outputs in the manner mentioned in the previous section.

It should be noted that in the calculation of the variance-covariance matrix ***R***, knowledge of the values of the means of the *f_i_* or *x_i_* are not required. While it is frequently stated that the variances of a distribution are independent of the position of the mean, in the RNL model this is a direct consequence of the implicit assumption that the variance of the noise, $e_{i}^{2}$, is independent of the quantum catch of receptor *i*. Hence the variances of the noise are independent of the means of the receptor outputs *f_i_* and the variances and covariances of the *x_i_* are independent of their means also.

Following a suggestion from a referee (whom we thank) we define, along with Vorobyev & Osorio [4], *δx_i_* and *δf_i_* by $\delta x_{i}=x_{i}-\langle x_{i}\rangle$ and $\delta f_{i}=f_{i}-\langle \;f_{i}\rangle$*.* Then $R_{ij}=\langle \delta x_{i}\delta x_{j}\rangle$, $e_{i}^{2}=\langle {\left(\delta f_{i}\right)}^{2}\rangle$ and, since the *f_i_* are independent, their covariances, $\left\langle \delta f_{i}\delta f_{j}\right\rangle$ with *i ≠ j*, are all zero.

Thus the diagonal terms of the matrix are

3.2 $$R_{ii}=\langle {\left(\delta x_{i}\right)}^{2}\rangle =\langle {\left(\delta f_{i}-\delta f_{i+1}\right)}^{2}\rangle =\langle {\left(\delta f_{i}\right)}^{2}-2\delta f_{i}\delta f_{i+1}+{\left(\delta f_{i+1}\right)}^{2}\rangle =e_{i}^{2}+e_{i+1}^{2}$$

and the only non-zero, off-diagonal terms are

3.3 $$R_{i,i+1}=R_{i+1,i}=\langle \delta x_{i}\delta x_{i+1}\rangle =\langle (\delta f_{i}-\delta f_{i+1})(\delta f_{i+1}-\delta f_{i+2})\rangle =-e_{i+1}^{2}.$$

In all the above expressions, the brackets ⟨⋯⟩ indicate averaging over the weighted values of the variable inside the brackets or both the variables if there are two.

To simplify the mathematical expressions below, from this point on we will write *v_i_* for $e_{i}^{2}$. We will return to the usual notation in the final expression.

The matrix ***R*** is a symmetric, tridiagonal matrix:

3.4 $$R=\left(\begin{array}{ccc} \begin{array}{ccc} v_{1}+v_{2} & -v_{2} & 0\\ -v_{2} & v_{2}+v_{3} & -v_{3}\\ 0 & -v_{3} & v_{3}+v_{4} \end{array} & \cdots & \begin{array}{cc} 0 & 0\\ 0 & 0\\ 0 & 0 \end{array}\\ \vdots & \ddots & \vdots \\ \begin{array}{ccc} 0 & 0 & 0\\ 0 & 0 & 0 \end{array} & \cdots & \begin{array}{cc} v_{N-2}+v_{N-1} & -v_{N-1}\\ -v_{N-1} & v_{N-1}+v_{N} \end{array} \end{array}\right).$$

The components of inverses of tridiagonal matrices are known to satisfy simple relationships [30]. The components of the inverse of the above matrix are given by

3.5 $$R_{ii}^{-1}=\frac{1}{\theta_{N-1}}\theta_{i-1}\emptyset_{i+1}\;i=1,2,\ldots N-1$$

and

3.6 $$R_{i,j}^{-1}=R_{j,i}^{-1}=\frac{1}{\theta_{N-1}}v_{i+1}\ldots v_{j}\theta_{i-1}\emptyset_{j+1}\;i=1,2,\ldots N-2,\;\;j=2,\ldots N-1,\;i<j.$$

In these expressions the *θ_i_* and $\emptyset_{i}\;$are functions of the *v_i_* that satisfy known recurrence relations.

The *θ_i_* satisfy the recurrence relation

3.7 $$\theta_{i}=(v_{i}+v_{i+1})\theta_{i-1}-v_{i}^{2}\theta_{i-2}$$

for *i* *=* 2, *… N *− 1 with initial values $\theta_{0}=1\;$and $\theta_{1}=\;v_{1}+v_{2}$.

Similarly the $\emptyset_{i}$ satisfy

3.8 $$\emptyset_{i}=(v_{i}+v_{i+1})\emptyset_{i+1}-v_{i+1}^{2}\emptyset_{i+2}$$

for i=N−2,…1 with initial values $\emptyset_{N}=1$ and $\emptyset_{N-1}=v_{N-1}+v_{N}$.

From equation (3.7), we have

3.9 $$\theta_{i}-v_{i+1}\theta_{i-1}=v_{i}(\theta_{i-1}-v_{i}\theta_{i-2}).$$

The term in brackets on the right side of the equation is the same as the expression on the left side with *i* replaced by *i *− 1*.* Repeated substitution of the bracketed term in terms of the next lower value of *i* gives eventually

3.10 $$\theta_{i}-v_{i+1}\theta_{i-1}=v_{i}v_{i-1}\ldots v_{2}(\theta_{1}-v_{2}\theta_{0})=v_{i}v_{i-1}\ldots v_{1}$$

or

3.11 $$\theta_{i}=v_{i+1}\theta_{i-1}+v_{i}v_{i-1}\ldots v_{1}.$$

Using *θ*_0_ = 1 and $\theta_{1}=\;v_{1}+v_{2}$ we have $\theta_{2}=v_{3}(v_{1}+v_{2})+v_{2}v_{1}$ and

3.12 $$\;\theta_{3}=v_{4}(v_{3}v_{1}+v_{3}v_{2}+v_{2}v_{1})+v_{3}v_{2}v_{1}.$$

It is clear that *θ_i_* is the sum of all possible products of *v*_1_, *v*_2_, … *v_i+_*_1_ with just one *v* missing. It can be written concisely as

3.13 $$\theta_{i}=v_{1}v_{2}\ldots .v_{i+1}\overset{i+1}{\underset{k=1}{\sum }}\frac{1}{v_{k}}.$$

Note that the determinant of *R**^−^*****^1^** is *θ_N_*_−1_ where

3.14 $$\;\theta_{N-1}=v_{1}v_{2}\ldots .v_{N}\overset{N}{\underset{k=1}{\sum }}\frac{1}{v_{k}}.$$

A similar reduction can be made for $\emptyset_{i}$. The variances involved are *v_i_* to *v_N_* giving

3.15 $$\emptyset_{i}=v_{i}v_{i+1}\ldots .v_{N}\overset{N}{\underset{k=i}{\sum }}\frac{1}{v_{k}}.$$

When these expressions are entered into equations (3.5) and (3.6) the products of the *v_i_* cancel, giving

3.16 $$R_{ii}^{-1}=\frac{\left(\sum_{k=1}^{i}(1/v_{k}))(\sum_{k=i+1\;}^{N}(1/v_{k})\right)}{\sum_{k=1}^{N}(1/v_{k})}$$

and

3.17 $$R_{i,j}^{-1}=R_{j,i}^{-1}=\frac{\left(\sum_{k=1}^{i}(1/\;v_{k}))(\sum_{k=j+1\;}^{N}(1/v_{k})\right)}{\sum_{k=1}^{N}(1/v_{k})}\;i<j.$$

In terms of these components, since $\Delta x_{i}=(\Delta f_{i}-\Delta f_{i+1})$, equation (3.1) is

3.18 $${\left(\Delta S\right)}^{2}=\overset{N-1}{\underset{i=1}{\sum }}R_{ii}^{-1}{\left(\Delta f_{i}-\Delta f_{i+1}\right)}^{2}+2\overset{N-1}{\underset{j=2}{\sum }}\overset{j-1}{\underset{i=1}{\sum }}R_{i,j}^{-1}(\Delta f_{i}-\Delta f_{i+1})(\Delta f_{j}-\Delta f_{j+1}).$$

## 4. Simplification

Vorobyev & Osorio [4] demonstrated for trichromatic and tetrachromatic vision that much simpler expressions could be obtained if (Δ*S*)^2^ is expressed in terms of all the ½*N*(*N *− 1) squared differences ${\left(\Delta f_{i}-\Delta f_{j}\right)}^{2}$. We will assume this is possible for any *N* and we will show that the result is a very simple formula.

Let

4.1 $${\left(\Delta S\right)}^{2}=\frac{1}{\sum_{k=1}^{N}(1/v_{k})}\overset{N-1}{\underset{i=1}{\sum }}\overset{N}{\underset{j=i+1}{\sum }}C_{i,j}{\left(\Delta f_{i}-\Delta f_{j}\right)}^{2}.$$

We can calculate the *C_i_*_,*j*_ by comparing the coefficients of $\Delta f_{i}^{2}$ and of $\Delta f_{i}\Delta f_{j}$ in the two expressions (3.18) and (4.1). We can take any values of *i* and *j* we choose as (Δ*S*)^2^ does not depend on which receptor is labelled *i* and which is *j.* For simplicity choose *i* *=* 1. In equation (3.18) the coefficient of $\Delta f_{1}^{2}$ is $R_{11}^{-1}$ where

4.2 $$R_{11}^{-1}=\frac{\left(\sum_{k=1}^{1}(1/\;v_{k}))(\sum_{k=2\;}^{N}(1/v_{k})\right)}{\sum_{k=1}^{N}(1/v_{k})}=\frac{\left(1/v_{1})(\sum_{k=2\;}^{N}(1/v_{k})\right)}{\sum_{k=1}^{N}(1/v_{k})}\;,$$

while the coefficient of $\Delta f_{1}^{2}$ in (4.1) is $(1/\sum_{k=1}^{N}(1/v_{k}))\sum_{j=2}^{N}C_{1,j}$. The two terms are equal if $C_{1,j}=\;1/v_{1}v_{j}$.

Similarly with *i* *=* 1 and *j* *=* 2 the coefficient of $\Delta f_{1}\Delta f_{2}$ in equation (3.18) is $2(R_{1,2}^{-1}-R_{1,1}^{-1}\;)$.

$R_{1,1}^{-1}$ is given by equation (4.2) and

4.3 $$R_{1,2}^{-1}=\frac{\left(\sum_{k=1}^{1}(1/\;v_{k}))(\sum_{k=3\;}^{N}(1/v_{k})\right)}{\sum_{k=1}^{N}(1/v_{k})}=\frac{\left(1/v_{1})(\Sigma_{k=3\;}^{N}(1/v_{k})\right)}{\Sigma_{k=1}^{N}(1/v_{k})}.$$

Hence

4.4 $$2(R_{1,2}^{-1}-R_{1,1}^{-1})=\frac{-2(1/v_{1})(1/v_{2})}{\sum_{k=1}^{N}(1/v_{k})},$$

which agrees with the coefficient of $\Delta f_{1}\Delta f_{2}$ in equation (4.1) if $C_{1,2}=1/v_{1}v_{2}$ . So the two expressions for (Δ*S*)^2^ are identical if $C_{i,j}=1/v_{i}v_{j}$. Consequently, we have that the square of the Mahalanobis distance between ***f*** and ***g*** for *N* receptors with (*N *− 1) opponent mechanisms is simply

4.5 $$(\Delta S){\;}^{2}=\frac{1}{\sum_{k=1}^{N}(1/e_{k}^{2})}\overset{N-1}{\underset{i=1}{\sum }}\overset{N}{\underset{j=i+1}{\sum }}\frac{{\left(\Delta f_{i}-\Delta f_{j}\right)}^{2}}{e_{i}^{2}e_{j}^{2}}.$$

We have replaced *v_i_* by $e_{i}^{2}\;$in this result.

This equation agrees with the results published by Vorobyev & Osorio [4] for dichromatic, trichromatic and tetrachromatic vision. To demonstrate this we will consider the trichromatic case as an example.

When *N* *=* 3 equation (4.5) is

4.6 $${\left(\Delta S\right)}^{2}=\frac{1}{\left(1/e_{1}^{2}+1/e_{2}^{2}+1/e_{3}^{2}\right)}\left(\frac{{\left(\Delta f_{1}-\Delta f_{2}\right)}^{2}}{e_{1}^{2}e_{2}^{2}}+\frac{{\left(\Delta f_{1}-\Delta f_{3}\right)}^{2}}{e_{1}^{2}e_{3}^{2}}+\frac{{\left(\Delta f_{2}-\Delta f_{3}\right)}^{2}}{e_{2}^{2}e_{3}^{2}}\right).$$

Multiplying both brackets by $e_{1}^{2}e_{2}^{2}e_{3}^{2}$ gives

4.7 $${\left(\Delta S\right)}^{2}=\frac{1}{\left(e_{2}^{2}e_{3}^{2}+e_{1}^{2}e_{3}^{2}+e_{1}^{2}e_{2}^{2}\right)}(e_{3}^{2}{\left(\Delta f_{1}-\Delta f_{2}\right)}^{2}+e_{2}^{2}{\left(\Delta f_{1}-\Delta f_{3}\right)}^{2}+e_{1}^{2}{\left(\Delta f_{2}-\Delta f_{3}\right)}^{2}).$$

Apart from reordering this is the same formula as given in [4].

## 5. Conclusion

Equation (4.5) gives (Δ*S*)^2^ for any number of receptor types. It can be simply stated as ${\left(\Delta f_{i}-\Delta f_{j}\right)}^{2}/e_{i}^{2}e_{j}^{2}$ summed over all pairs of receptors divided by $1/e_{i}^{2}$ summed over all receptors. This equation allows the calculation of RNL colour distances for *n*-chromatic visual systems, facilitating the easy application of this model to investigate colour discrimination in organisms with complex visual systems and unknown chromatic mechanisms (c.f. [14,19]).

A key premise of the RNL model is that the discriminability of colour stimuli is set by receptor noise and not opponent mechanisms (assumption 3), and as such the way in which receptor responses are combined within opponent mechanisms (the matrix ***F*** in equation (2.3)) does not determine the discriminability of colour signals (e.g. [11]). However, we point out that the form of equation (4.5) depends critically on equation (2.4), which itself is determined by assumption 2. Hence in the RNL model, opponent mechanisms are not completely unspecified because their choice is restricted by assumption 2.

## Data accessibility

This article has no additional data.
